# Characterisation of Mesenchymal Stromal Cells (MSCs) from Human Adult Thymus as a Potential Cell Source for Regenerative Medicine

**DOI:** 10.3390/jcm14103474

**Published:** 2025-05-15

**Authors:** Martina Ramsperger-Gleixner, Chang Li, Nina Wallon, Annika Kuckhahn, Volker Weisbach, Michael Weyand, Christian Heim

**Affiliations:** 1 Department of Cardiac Surgery, University Hospital of Erlangen-Nürnberg, Krankenhausstraße 12, 91054 Erlangen, Germany; 2 Friedrich-Alexander Universität Erlangen-Nürnberg, 91054 Erlangen, Germany; 3 Department of Transfusion Medicine and Haemostaseology, University Hospital of Erlangen-Nürnberg, Krankenhausstraße 12, 91054 Erlangen, Germany; 4 Department of Cardiac and Vascular Surgery, Klinikum Bayreuth GmbH, Medizincampus Oberfranken (MCO), of Friedrich-Alexander University Erlangen-Nürnberg, Preuschwitzer Straße 101, 95445 Bayreuth, Germany

**Keywords:** mesenchymal stromal cell, adipose tissue, adult thymus, platelet lysate, cell therapy, cardiac surgery

## Abstract

**Background:** Mesenchymal stem cell-based therapy may be indicated in ischaemic heart disease. The use of autologous adipose-derived mesenchymal stromal cells (AdMSCs) offers regenerative potential due to their paracrine effects. The aim of this study was to expand and characterise adult human thymus-derived MSCs harvested during open heart surgery with respect to their stem cell and paracrine properties. **Methods**: Enzymatically and non-enzymatically isolated human thymic AdMSCs (ThyAdMSCs) were cultured in xeno-free media containing pooled human platelet lysate (pPL). MSC characterisation was performed. Ex vivo expanded ThyAdMSCs were differentiated into three lineages. Proliferative capacity and immunomodulatory properties were assessed by proliferation assays and mixed lymphocyte reaction, respectively. Gene expression analysis was performed by qPCR. **Results:** Both isolation methods yielded fibroblast-like cells with plastic adherence and high proliferation. Flow cytometry revealed distinct expression of MSC markers in the absence of haematopoietic cell surface markers. Ex vivo expanded ThyAdMSCs could be differentiated into adipocytes, osteocytes, and chondrocytes. Activated peripheral blood mononuclear cells were significantly reduced when co-cultured with ThyAdMSCs, indicating their ability to inhibit immune cells in vitro. Gene expression analysis showed significantly less IFNγ and TNFα, indicating an alteration of the activated and pro-inflammatory state in the presence of ThyAdMSCs. **Conclusions**: These results demonstrate an efficient method to generate AdMSCs from human thymus. These MSCs have a strong immunomodulatory capacity and are, therefore, a promising cell source for regenerative medicine. The culture conditions are crucial for cells to proliferate in culture. Further research could explore the use of ThyAdMSCs or their secretome in surgical procedures.

## 1. Introduction

Human mesenchymal stromal cells (MSCs) have been used as a cell source for regenerative medicine for some time, mainly because of their regenerative and immunomodulatory properties [1,2]. The nomenclature “MSCs” usually includes heterogeneous multipotent mesodermal stromal and progenitor cells up to mature stromal cells cultured ex vivo. Concerning their biological and regenerative properties and the multitude of different names, one group of scientists has proposed changing the name of mesenchymal stem cells to “medicinal signaling cells” [3,4], although this has not yet been adopted. Others postulate that only cells from a specific stromal component of the bone marrow microenvironment should be termed “mesenchymal stem cells”. MSCs from other tissue sources should be termed to as “multipotent mesenchymal stromal cells” [5].

Adipose tissue, mainly isolated from bone marrow, umbilical cord blood, and placenta, is another important source of MSCs [6]. Adipose-derived mesenchymal stromal cells (AdMSCs) are of particular interest because AdMSCs are easily obtained from a variety of adipose tissues and meet the minimal criteria for the definition of multipotent mesenchymal stromal cells [7,8]. The wide range of methods used to isolate adipose tissue, such as liposuction and lipectomy, and the different origins result in variable cell numbers and cells with varying degrees of multipotency.

The use of MSCs as a therapeutic option is widespread and covers almost all medical fields [4]. For example, there are several animal studies and also clinical trials using MSCs to heal chronic wounds, particularly in lower extremity ulcers, radiation burns, and pressure ulcers [9]. In cardiac surgery, deep sternal wound infection (DSWI) is a serious complication after median sternotomy. The incidence of chronic wounds after cardiac surgery ranges from 0.5% to 6.8%, and the resulting in-hospital mortality of DSWI varies from 7% to 35% [10]. Therefore, the supportive use of autologous MSCs for DSWI may be a promising regenerative therapy. Another potential area for the use of MSCs as a cell therapy is ischaemic heart disease, for example, after a myocardial infarction (MI). Several approaches to cardiac regeneration have been studied in clinical trials. They have shown that cell therapy, especially with MSCs, is safe, but there is still room for improvement in terms of efficacy [11].

Adult thymic adipose tissue has received little attention as a source of MSCs for regenerative therapies. The thymus develops during embryonic development and undergoes a transformation in adulthood, during which it decreases in size and the glandular tissue is replaced by adipose tissue [12]. During open-heart surgery, the thymus is usually resected and discarded after median sternotomy to improve surgical exposure. Since the origin, isolation method and culture conditions influence the proliferation and differentiation capacity as well as the content of biologically active molecules in MSCs, it is important to precisely characterise the isolated and ex vivo expanded cells in detail. The aim of this study was, therefore, to isolate and characterise AdMSCs from the adult thymus. In addition, we established animal component-free cell culture conditions and tested the immunomodulatory capacity of the isolated thymic cells with a view to future applications in cell therapy

## 2. Materials and Methods

### 2.1. Ethics

Thymus tissue was obtained from patients undergoing open heart surgery in accordance with the Ethics Committee of the University of Erlangen-Nürnberg (Ethics Approval 338_16B) and with the informed consent of the patients.

### 2.2. Isolation of AdMSCs from Adult Thymus

AdMSCs were isolated from adult thymus directly after tissue harvest from the operating theatre. Samples were collected in phosphate-buffered saline (PBS) supplemented with penicillin/streptomycin (pen/strep) (100 U/100 µg; Biochrom AG, Berlin, Germany). Thymic tissue (6–7 mL) was washed 6 times in PBS after removal of damaged areas and major vessels. After a subsequent centrifugation step (720 g for 3 min at room temperature), the tissue was divided into two pieces for different isolation methods, namely (1) enzymatic digestion and (2) outgrowth.

Method (1): Thymic tissue (~4 mL) was cut into small pieces, incubated in an equal volume of collagenase I (0.1% *w*/*v* solution in PBS without Ca^2+^, Mg^2+^) for 90 min at 37 °C with gently stirring. After two centrifugation steps (10 min, 4 °C, 400 g and 5 min, 20 °C, 400 g) and removal of the supernatants, the stromal vascular fraction (SVF) was resuspended in 10 mL DMEM (Lonza, Switzerland) with pen/strep (100 U/100 µg; Biochrom AG, Germany). The cell suspension was filtered through a 100 µm and a 40 µm cell strainer (Falcon), centrifuged (5 min, 20 °C, 400 g), and resuspended in DMEM containing pen/strep, 2 IU/mL heparin-sodium-25.000 (Ratiopharm, Ulm, Germany), and 10% pooled human platelet lysate (pPL; Department of Transfusion Medicine and Haemostaseology, University Hospital of Erlangen-Nürnberg, Germany). Finally, cells were cultured on uncoated plastic at a density of 2.5 × 10^5^ cells/cm^2^ in a humidified atmosphere containing 5% CO_2_ at 37 °C.

Method (2): The remaining tissue was cut into 3–5 mm^2^ pieces, transferred to culture plates (10 pieces/9.6 cm^2^), and cultured in 3 mL αMEM (Lonza, Basel, Switzerland) per well, again supplemented with pen/strep, huPL, and heparin at the same concentrations as in Method 1. Cultivation was performed in a humidified atmosphere of 5% CO_2_ at 37 °C without agitation for at least 3 days.

### 2.3. Animal Component-Free Cell Culture

For future adequate stem cell application, isolated AdMSCs need to be expanded ex vivo. The important property of MSCs to adhere to plastic requires the use of trypsin-EDTA. To avoid animal components, we used recombinant trypsin-EDTA (Biological Industries, Kibbutz Beit Haemek, Israel) and as stop solution soybean trypsin inhibitor (Biological Industries, Israel) at concentrations described in the manufacture’s protocol. As a growth factor supplier, we added 10% pooled human platelet lysate (pPL) to the medium. This was obtained from expired platelet apheresis concentrates from healthy volunteers at the Department of Transfusion Medicine and Haemostaseology at the University Hospital of Erlangen, Germany.

### 2.4. Characterisation of AdMSCs from Adult Thymus

Flow cytometry was used to identify cell surface markers of ThyAdMSCs (BD FACS Verse Flow Cytometer, Becton Dickinson, Franklin Lakes, NJ, USA). Nearly confluent cell cultures (80%) were harvested and adjusted to 1000 cells/µL PBS. Antibodies and reagents were used according to the manufacturer’s recommendations and are listed in Table 1.

### 2.5. In Vitro Multilineage Differentiation and Histological Verification

Multilineage differentiation of ThyAdMSCs into adipocytes, chondrocytes, and osteocytes was performed using specific differentiation media according to the manufacturer’s instructions. The StemPro Adipogenesis Differentiation Kit (Gibco, ThermoFisher Scientific, Schwerte, Germany) was used for adipocyte differentiation. For histological examination, the fatty acids were stained with Oil Red O (Sigma-Aldrich, Taufkirchen, Germany) after cultivation in the appropriate media (after approximately two weeks). Chondrocytes were differentiated in ChondroMax Differentiation Medium (Sigma-Aldrich, Taufkirchen, Germany) using micromass culture. Briefly, 10 µL drops of cell suspension (1.6 million cells/mL) were pipetted into 24-well culture plates and incubated for 2 h at 37 °C in a humidified atmosphere containing 5% CO_2_. The wells were filled with 1 mL of differentiation medium. After 3 to 4 weeks, the wells were carefully rinsed with PBS, the cells were fixed with Roti-Histofix 4% (Roth Ltd, Nürnberg, Germany) for 30 min at room temperature and washed again with PBS. Before staining with Alcian Blue, the cells were incubated with 3% acetoacetic acid for 5 min. The cells were then stained with Alcian Blue solution (pH 1–1.5) for 30 min at room temperature, followed by a wash step with 3% acetoacetic acid and another with distilled water.

Osteogenic differentiation was carried out with MesenCult^TM^ Osteogenic Stimulatory Kit (StemCell Technologies, Köln, Germany) in 12-well culture plates (2500 cells/well). After 14 days of cultivation in this medium, an Alizarin Red staining was performed. The cells were washed with PBS and fixed with 70% ethanol for 10 min. After washing with distilled water, the cells were incubated with 1.25% Alizarin water solution (adjusted to pH 4.1 with 0.5% ammonium hydroxide) for 10 min and then washed several times with distilled water.

All image analyses were performed on a colour monitor using cellSens 1.18 software (Olympus, Hamburg, Germany).

### 2.6. Proliferation Assay

Cell proliferation assays were carried out in 96-well culture plates. For this purpose, ThyAdMSCs were seeded in triplicate at 20,000, 10,000, 5000, 2500, and 1250 cells/well and incubated overnight in the appropriate media described above. The next day (day 1), the media was discarded, and the cells were incubated in PrestoBlue^TM^ Cell Viability Reagent (Invitrogen^TM^; ThermoFisher Scientific, Schwerte, Germany) for 2 h according to the manufacturer’s instructions. Measurements with PrestoBlue^TM^ Cell Viability Reagent were repeated on days 4 and 7 after seeding. Cells were returned to their respective culture media between readings. Photometric readings were taken using a multiscan microplate photometer (Thermo Scientific^TM^; ThermoFisher Scientific, Schwerte, Germany) at 560 and 620 nm.

### 2.7. Mixed Lymphocyte Reaction and Indolamine-2,3-Dioxygenase (IDO) Quantification

To investigate the immunomodulatory effect of ThyAdMSCs, we conducted a mixed lymphocyte reaction (MLR). Peripheral blood mononuclear cells (PBMCs) were isolated from heparinised blood of healthy volunteers by Lymphoprep^TM^ density gradient (StemCell Technologies, Köln, Germany). PBMCs were resuspended in RPMI 1640 (Gibco Fisher Scientific) containing 10% FCS, 1% L-glutamate, 1% pen/strep, and 1% sodium pyruvate and cultured for 3 h at 37 °C in a humidified atmosphere containing 5% CO_2_. Meanwhile, cultured ThyAdMSCs were treated with mitomycin C (MSM) (10 µg/mL) (StemCell Technologies) to stop their proliferation without killing them. MLR was carried out with activated (PBac) (human CD3/CD28 T cell activator 25 µL/mL, StemCell Technologies) and non-activated PBMCs (PBMC) in 96-well culture plates (for proliferation analysis) or in 24-well culture plates (for IDO measurements). Cells were seeded at a ratio of 1 (MSM): 8 (PBac) in triplicate for each measurement. After culturing, supernatants (with PBac) and adherent ThyAdMSCs (MSM) were collected separately on days 4, 6, and 7 for gene expression analysis or IDO quantification by ELISA (DuoSet ELISA, R&D Systems Ltd, Wiesbaden, Germany). As the ELISA samples had to be lysed before measurement, we performed three freeze–thaw cycles (physical lysis). Each measurement was performed in triplicates according to manufacturer’s instructions.

### 2.8. Quantitative Gene Expression Analysis

For gene expression analysis of PBMCs from the MLR, the supernatant containing the PBMCs (PBac and activated PBMCs co-cultured with MSM = CoPB) was collected and centrifuged (5 min, 9300 g, RT), the pellet was snap frozen in liquid nitrogen and stored at −80 °C. The mRNA isolation and complementary DNA synthesis were performed according to standard protocols. Adherent ThyAdMSCs (MSM and MSM co-cultured with PBac = CoMS) were detached with RLT buffer (RNeasy Lysis Buffer, Qiagen, Hilden, Germany), and subsequent mRNA isolation and complementary DNA synthesis were also carried out according to standard protocols. Quantitative RT-PCR was performed in triplets using the StepOne Real-Time PCR System and the TaqMan Gene Expression Master Mix (Applied Biosystems, Forster City, CA, USA). Each PCR product was cloned into a TOPO cloning vector (Invitrogen, Karlsruhe, Germany) to generate PCR standards. Cloned amplicons were verified by sequence analysis. Standard curves with known concentrations of template copy numbers were used to detect the expression of the amplified target gene. Probes were normalised against the expression of the housekeeping gene 18S rRNA. Results are given as relative copy numbers. Primer and probe sequences are published in a previous study [13]. All oligonucleotides were synthesised by Eurofins Genomics (Ebersberg, Germany).

### 2.9. Statistical Analysis

Results are presented as mean per group ± standard deviation. The data were subjected to statistical analysis using a one-way Brown-Forsythe and Welch ANOVA, conducted with GraphPad Prism 10.2.0 A *p*-value of ≤0.05 was considered statistically significant.

## 3. Results

### 3.1. Successful Isolation and Cultivation of ThyAdMSCs Under Animal Component-Free Culture Conditions

Thymic tissue was obtained from 17 adult patients (aged between 37 and 81 years; mean 62.1 years) undergoing open heart coronary artery bypass graft or valve surgery in our department. The randomly selected patients included 6 women and 11 men, of whom only one was overweight and two were obese. Three had type 2 diabetes mellitus, but none was insulin dependent.

**Figure 1:**
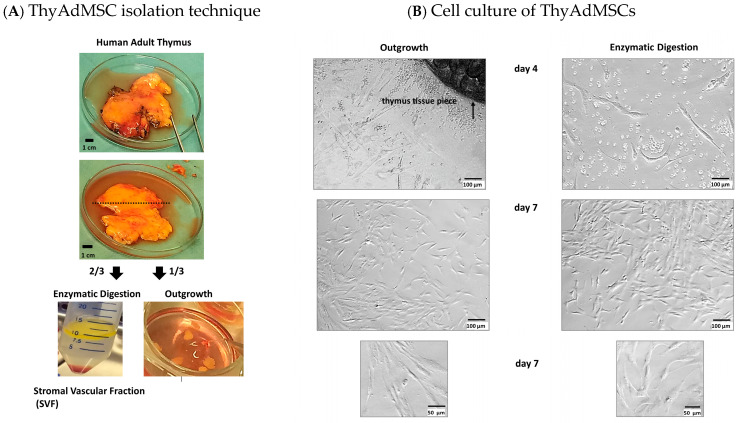
Isolation of mesenchymal stromal cells from adult thymus adipose tissue. (**A**) After “cleansing”, the tissue was divided for two different isolation methods: two-thirds of the tissue was used for collagenase I enzyme digestion to obtain the stromal vascular fraction, which was seeded into culture plates. The other third of the tissue was cut into small pieces of ~3–5 mm^2^ to allow the outgrowth of MSCs (explant culture). (**B**) Morphology of ThyAdMSCs after 4 and 7 days, respectively.

We successfully isolated ThyAdMSCs from all 17 thymus samples by enzymatic digestion and outgrowth (Figure 1A shows the isolation procedure). The cells were cultured under animal-free culture conditions with standard media DMEM or αMEM containing 10% pooled platelet lysate (pPL) from the local Department of Transfusion Medicine and Haemostaseology. We typically observed the first cells to spread from the thymus pieces on day 4 (outgrowth method Figure 1B). After five to six days, we removed the pieces of tissue from the outgrowth cultures and rinsed the cells with warm PBS. Cells from both isolation methods were cultured in a humidified atmosphere of 5% CO_2_ at 37 °C, and the media was changed every third day. Both isolation methods resulted in well proliferating cells with fibroblast-like morphology and plastic adhesion with homogeneous distribution in the wells (Figure 1B).

### 3.2. Characterization of ThyAdMSCs

**Figure 2:**
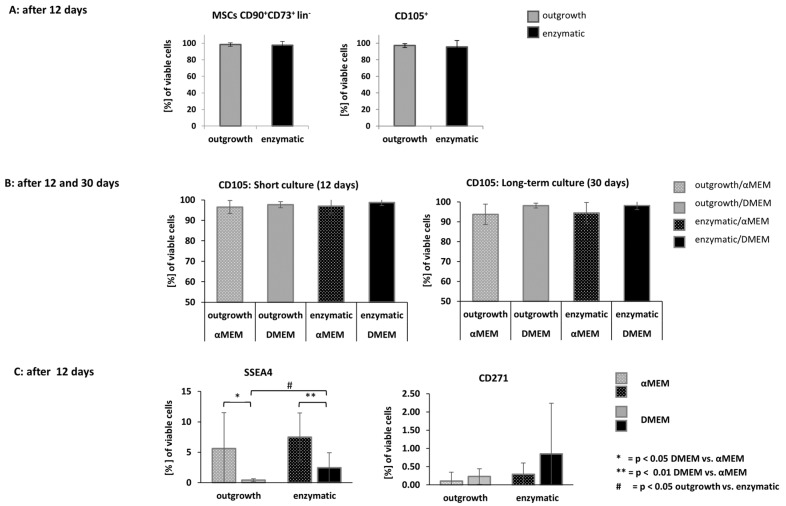
Flow cytometry of ThyAdMSCs cultured in αMEM or DMEM with 10% pPL and pen/strep. (**A**) Expression of positive stem cell markers CD90, CD73, and CD105 and negative markers Lin (=CD11b, CD19, CD31, CD34, CD45, CD144, and HLA-DR) in “outgrowth” ThyAdMSCs cultured in αMEM and “enzymatic” MSCs grown in DMEM (n = 10). (**B**) Surface expression of CD105 as a function of medium and culture time (n = 5). (**C**) Surface markers SSEA4 and CD271 as a function of isolation method and culture medium. The values are given as [%] of viable cells.

To confirm that the isolated cells from adult thymus fulfil the main criteria for mesenchymal stromal cells, we used flow cytometry to determine the surface markers defined by the ISCT (International Society for Cellular Therapy) [7]. ThyAdMSCs from both isolation methods showed a consistent pattern in multiparameter flow cytometry after short-term cultivation (typically after 12 days of cultivation this means passage 2 or 3) at 80–90% confluence. As expected, the isolated ThyAdMSCs were negative for the surface markers CD11b, CD19, CD31, CD34, CD45, CD144, and HLA-DR (summarised as “lin-”) and positive for CD73, CD90 and CD105 (all ˃ 95%) (Figure 2A). Flow cytometry was performed on fresh and cryopreserved ThyAdMSCs. This showed no difference in the expression of surface markers. A target dose of 100–150 million cells is usually required for a cell therapy. This could be achieved in approximately 3–4 weeks. Accordingly, we also analysed long-term culture of ThyAdMSCs. Interestingly, the proportion of CD105 on cells obtained by outgrowth is slightly reduced in long-term cultures (expression range 88.50–99.90%). To determine the reason for this CD105 loss, we compared cells cultured in DMEM or αMEM. We found that the CD105 loss occurred only in cells cultured in αMEM, and only after 30 days (cell culture at passage 5 or 6), but not in DMEM, regardless of the isolation method [long-term culture: 90.9% ± 5.10 (αMEM) vs. 98.1% ± 1.23 (DMEM); *p* = 0.376 (outgrowth) and 94.5% ± 5.23 (αMEM) vs. 98.1% ± 2.07 (DMEM); *p* = 0.642 (enzymatic); (n = 5 per group)] (Figure 2B). However, this difference in CD105 surface expression did not reach significance. Expression of CD90, CD73, and lin- markers was unchanged at both time points; (n = 5 per group). The additional analyses of the surface proteins SSEA4 and CD217 (at day 12) showed, above all, that both surface markers have a broad spectrum of expression. Again, a difference was observed between the media used. For both isolation methods, we found a higher expression of SSEA4 in cells grown in αMEM compared to cells cultured in DMEM [outgrowth: 5.61% ± 5.91 (αMEM) vs. 0.40% ± 0.24 (DMEM); *p* = 0.024 and enzymatic: 7.50% ± 3.96 (αMEM) vs. 1.60% ± 2.46 (DMEM); *p* = 0.022) (Figure 2C). A significant difference was also observed when comparing SSEA4 levels between cells gained by the outgrowth method and cultured in DMEM and those obtained by the enzymatic method and cultured in DMEM (Figure 2C). This discrepancy was not observed for CD271 expression, which showed a high standard deviation and low expression in all experimental groups (Figure 2C).

### 3.3. Proliferation Capacity Depending on the Culture Medium

**Figure 3:**
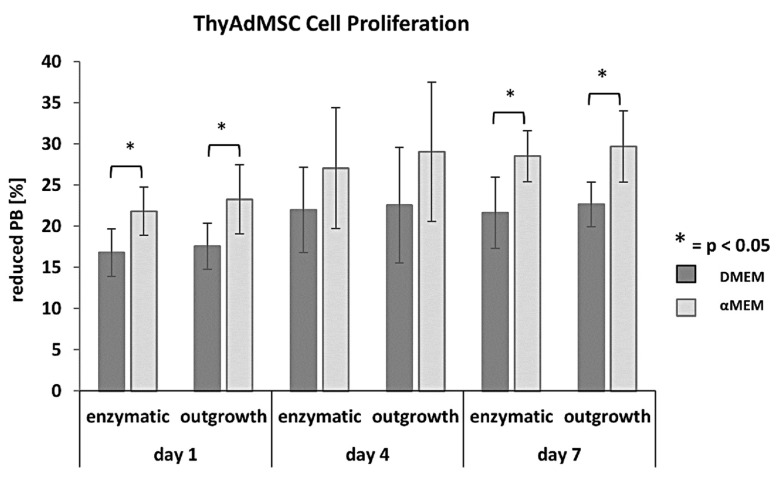
ThyAdMSCs proliferation assay using PrestoBlue^®^. For cell proliferation analysis at different time points, media was discarded, and cells were incubated in PrestoBlue^TM^ Cell Viability Reagent (Invitrogen^TM^; ThermoFisher) for 2 h according to the manufacturer’s instruction. Photometric measurements were performed at 560 and 620 nm. The values are given in [%] of reduced PrestoBlue (PB). PrestoBlue is a non-toxic, resazurin-based solution that exploits the ability of living cells to convert resazurin to the fluorescent molecule resorufin. The resorufin can be detected photometrically. It is used to measure the amount of PrestoBlue reagent metabolized during incubation and is an indicator of the amount of metabolically active cells present in the culture. The proliferation assays presented in Figure 3 are based on 20,000 cells/well seeded on day 0. The assay was performed in triplicate at each time point (n = 5; * = *p* < 0.05).

Based on the above results, we performed proliferation studies to compare media and isolation methods, using the PrestoBlue^®^ proliferation assay in 96-well culture plates with cells of passage 2 or 3. These proliferation analyses on days 1, 4 and 7 also revealed a difference in the ability of ThyAdMSCs to proliferate depending on whether they were cultured in αMEM or DMEM (Figure 3). Cells grown in DMEM had a significantly lower proliferation capacity than cells grown in αMEM. This was most evident in experiments where 20,000 cells were seeded per well at the beginning (day 0). The differences were significant on days 1 and 7 [Figure 3: day 1 enzymatic: 16.78% ± 2.86 (DMEM) vs. 21.82% ± 2.93 (αMEM); *p* = 0.025; day 1 outgrowth: 17.56% ±2.80 (DMEM) vs. 23.27% ± 4.17 (αMEM); *p* = 0.039 and day 7 enzymatic: 21.62% ± 4.34 (DMEM) vs. 28.53% ± 3.10 (αMEM); *p* = 0.041; day 7 outgrowth: 22.65% ± 2.71 (DMEM) vs. 29.66% ± 4.33 (αMEM); *p* = 0.040; n = 5 per group].

### 3.4. ThyAdMSCs Are Capable of Multilineage Differentiation

**Figure 4:**
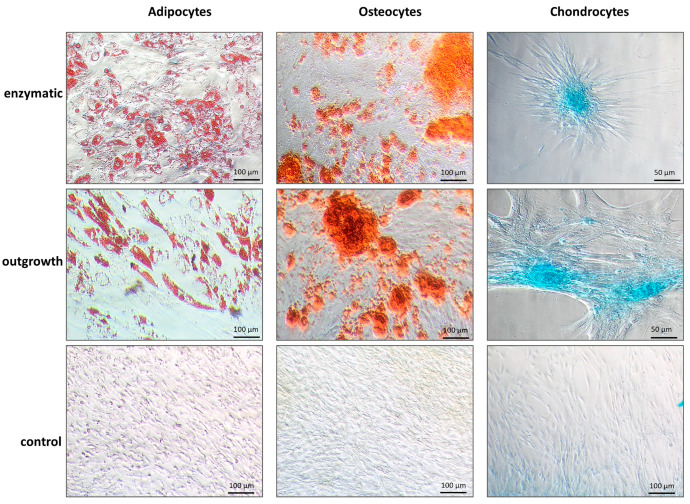
In vitro multilineage differentiation of ThyAdMSCs. Representative images of ThyAdMSCs differentiating into adipocytes (Oil Red O staining), osteocytes (Alizarin Red staining), or chondrocytes (Alcian Blue staining) under specific culture conditions. ThyAdMSCs cultured in DMEM or αMEM were used as controls.

To test whether ThyAdMSCs could differentiate into osteoblasts, adipocytes, and chondroblasts in vitro, the cells (passage 2 or 3) were cultured in specific differentiation media according to the manufacture’s instruction. The ThyAdMSCs showed multilineage differentiation capacity as shown in Figure 4. This multilineage differentiation capacity was independent of the primary isolation method and the medium in which the cells were initially cultured.

### 3.5. ThyAdMSCs Inhibit Immune Cell Proliferation in Mixed Lymphocyte Reaction

**Figure 5:**
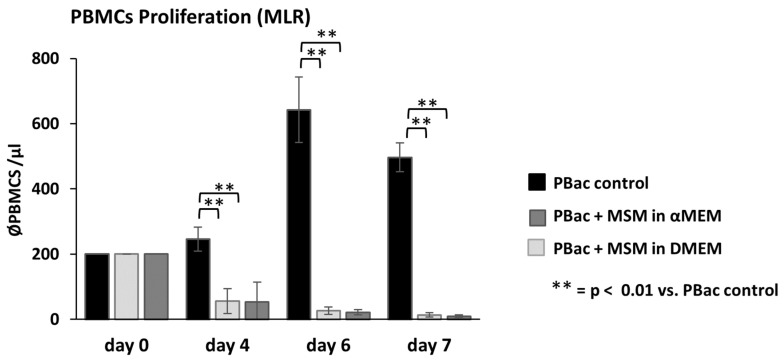
ThyAdMSCs in mixed lymphocyte reaction with activated PBMCs. MLR was carried out with activated PBMCs (PBac) and mitomycin C-treated ThyAdMSCs (MSM) in 96-well plates at a ratio of 1 (MSM): 8 (PBac). The number of PBMCs present in the cell culture was counted on days 4, 6, and 7 using a Neubauer grating haemocytometer and expressed as the mean value per µL (n = 4, ** = *p* < 0.01.) All measurements were carried out in triplicate.

The immunomodulatory properties of MSCs are a key feature for their use in regenerative cell therapies. To demonstrate that ThyAdMSCs also affect immune cell proliferation, we performed mixed lymphocyte reactions (MLR) with activated human PBMCs-(PBac) co-cultured with ThyAdMSCs (of passage 2–4) (mitomycin C-treated, MSM). Freshly isolated PBMCs were activated with CD3/CD28 T cell activator and co-cultured (200 cells/µL) with MSM. Activated PBMCs (PBac) without MSM served as a control. Cell count analysis on days 4, 6, and 7 showed a high proliferation rate of PBac in the control group with a maximum on day 6. This was significantly blocked when PBac were co-cultured with mitomycin C-treated ThyAdMSCs (CoPB) (irrespective of the culture media) (Figure 5) [day 4 average cell count/µL: 56.22 (PBac + MSM in αMEM), 53.60 (PBac + MSM in DMEM), vs. 246.03 (PBac control); day 6: 26.26 (PBac + MSM in αMEM), 21.83 (PBac + MSM in DMEM) vs. 642.67 (PBac control) and day 7: 13.33 (PBac + MSM in αMEM), 9.45 (PBac + MSM in DMEM) vs. 496.83 (PBMC control); all co-culture groups are *p* < 0.01 vs. control]. These results indicate a strong immunomodulatory effect of ThyAdMSCs on peripheral blood mononuclear cells.

### 3.6. Altered Gene Expression of Activated PBMCs Co-Cultured with ThyAdMSCs

**Figure 6:**
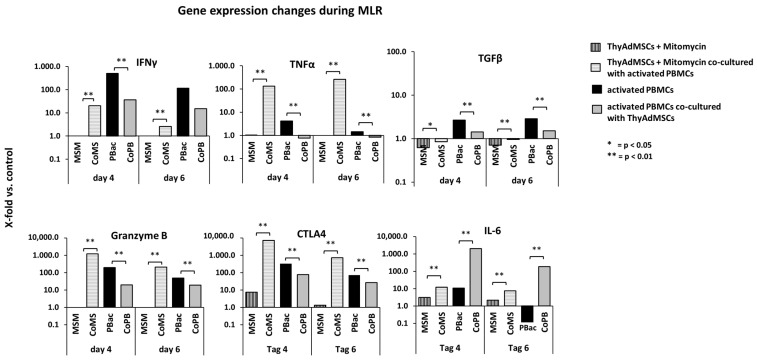
Gene expression under MLR conditions. For the gene expression studies we used cultured ThyAdMSCs without mitomycin C as the baseline for ThyAdMSCs with mitomycin C (MSM) and ThyAdMSCs with mitomycin co-cultured with activated PBMCs (CoMS). Non-activated PBMCs were used as the control group for activated PBMCs (PBac) or activated PBMCs co-cultured with mitomycin-treated ThyAdMSCs (CoPB). Expression was measured by quantitative RT-PCR and is stated as x-fold change relative to the corresponding control (* = *p* < 0.05; ** = *p* < 0.01).

To learn more about the influence of ThyAdMSCs on activated PBMCs, we performed an MLR and determined the expression of inflammatory genes in PBMCs and ThyAdMSCs on day 4 and 6. For these gene expression studies we used ThyAdMSCs that were split between 3 and 4 times. First, we compared the gene expression of ThyAdMSCs obtained by different isolation methods and cultured in different media. No differential expression profile could be determined between these groups. Therefore, we pooled several gene expression analyses, as shown in Figure 6 (n = 4). Expression was given as the x-fold change compared to the corresponding control. Specifically, we took the gene expression of cultured ThyAdMSCs without mitomycin C treatment as baseline and compared it to the values of ThyAdMSCs + mitomycin C (MSM) and ThyAdMSCs + mitomycin C + activated PBMCs (CoMS). In addition, the gene expression of non-activated PBMCs was used as a baseline for activated PBMCs (PBac) and activated PBMCs that were cultured together with ThyAdMSCs treated with mitomycin C (CoPB). In ThyAdMSCs cultured with PBac (CoMS), the expression of IFNγ, TNFα, granzyme B, CTLA4, IL-6, and TGFβ increased massively compared to ThyAdMSCs (MSM) without PBac on day 4. This was also observed on day 6, with a slight decrease in the expression of IFNγ, granzyme B, CTLA4, and IL-6, and a tendency to increase that of TNFα and TBFβ (Figure 6). At the same time, the expression of these genes in PBMCs drastically decreases on day 4 and 6 when they are cultured together with ThyAdMSCs compared to PBac without ThyAdMSCs. Interestingly, however, the expression of IL-6 in CoPB was massively increased compared to PBac alone at both time points (Figure 6).

### 3.7. IDO Activity During MLR

**Figure 7:**
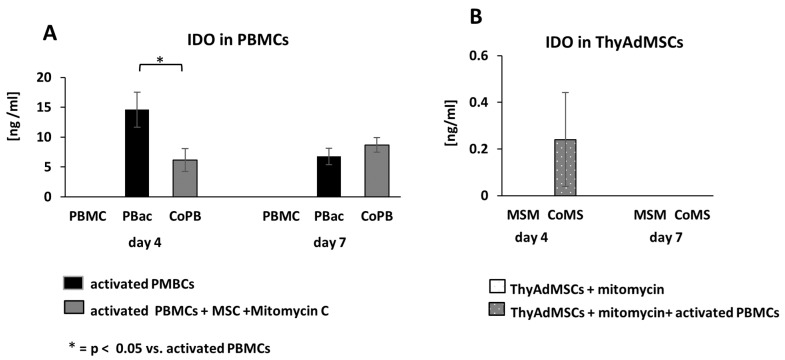
IDO production by PBMCs and ThyAdMSCs during MLR. For IDO detection ELISA, cells were seeded in triplicate in 24-well plates at a ratio of 1 (MSM): 8 (PBac) (n = 3). After culturing, supernatants (containing PBMCs) (**A**) and adherent ThyAdMSCs (**B**) were collected separately on days 4 and 7 for quantification of the IDO levels by ELISA. Three freeze–thaw cycles were performed before samples were used for ELISA. The IDO measurement was performed in triplicate according to manufacturer’s instructions.

The tryptophan-degrading enzyme IDO acts as a rate-limiting enzyme in the kynurenine pathway. It induces the breakdown of tryptophan, an amino acid required for cell proliferation, thereby inhibiting T cell proliferation [14]. We quantified the IDO production by ThyAdMSCs (passage 3–4) and PBMCs during MLR by ELISA. ThyAdMSCs from both isolation methods (2 each) were used for MLR. There was no difference between the methods regarding the immunomodulatory effect of the ThyAdMSCs, so these data were combined. ThyAdMSCs for MLR were cultured in αMEM. As shown in Figure 7A, IDO is not measurable in non-activated PBMCs (PBMC), but it is produced in large amounts in activated PBMCs (PBac) after 4 days [14.62 ng/mL ± 2.93] (Figure 7A), confirming the elevated activation status. When PBac are co-cultured with MSM (CoPB), IDO is significantly reduced on day 4 [6.16 ng/mL ± 1.91, *p* = 0.038] compared to PBac. On day 7, the IDO levels decrease to similar levels in both groups [6.78 ng/mL ± 1.38 (PBac) vs. 8.70 ng/mL 1.25 (CoPB), *p* = 0.141]. Looking at IDO production in ThyAdMSCs (Figure 7B), IDO can only be measured in MSCs cultured with PBac (CoMS) on day 4. On day 7 no IDO is detectable in either MSC group (Figure 7B).

## 4. Discussion

Cellular therapy with ex vivo expanded mesenchymal stromal cells offers a promising treatment option for several inflammatory or even ischaemic diseases. There are numerous approaches to use these cells for regenerative medicine [4,15,16]. However, the production of MSC suspensions is very heterogeneous, and parameters such as tissue origin, isolation method, and culture conditions are crucial as these parameters influence the “behavior” of the MSCs. Therefore, it is essential to characterise each procedure for obtaining MSCs with a view to their future application.

In the present study, we used two different isolation methods and two standard media to determine the possible advantages of one method over the other for the isolation of adult thymic MSCs. Following Klein and colleagues [17], we used an explant culture method in which the MSCs were initially cultured in αMEM. In addition, we used the most commonly practiced collagenase digestion and expanded the cells in DMEM. In both cases, the respective medium was supplemented with 10% pPL. Each method yielded well proliferating cells with fibroblast-like morphology, plastic adhesion [7], and an acceptable cell yield. Using flow cytometry analysis, we observed that the expression of the surface marker CD105 decreased during the long-term culture of ThyAdMSCs. The level of SSEA4, which we additionally measured as a marker for pluripotent cells from adipose tissue, also changed [18]. Contrary to the descriptions in the literature [19,20,21] and our own assumptions, this was not due to the isolation method. When ThyAdMSCs were cultured in αMEM for a longer period of time, they showed a slightly reduced CD105 surface expression and significantly increased SSEA4 expression, but not when the cells were cultured in DMEM. However, this phenomenon did not occur within the first two weeks of culture. On the other hand, ThyAdMSCs cultured in αMEM showed a significantly higher proliferation rate than cells grown in DMEM, while there was only a marginal difference between the isolation methods. These results are in contrast with other studies in which the medium had no effect on the expression of typical surface makers, but explant protocols resulted in higher proliferation rates compared to enzymatically obtained MSCs [19,20,22]. Overall, however, it is difficult to compare individual studies as there are always differences in the methods and culture conditions that cannot be compared with each other. Therefore, the establishment of precise standard protocols is desirable. Interestingly, in our study neither the media nor the isolation procedure had any influence on the differentiation into osteoblasts, adipocytes and chondroblasts in vitro.

A very important aspect in the preparation of MSC suspensions for cell therapy is the tissue origin used for ex vivo expansion of MSCs to achieve a desired target dose of 100–150 million cells [23]. We avoided the use of animal components to detach the adherent cells and used human pooled platelet lysate from the local Department of Transfusion Medicine and Haemostaseology (instead of fetal calf serum). This ensured that the MCSs were supplied with sufficient proliferation-promoting growth factors. The aim of avoiding xenogeneic components was to prevent potential anti-human immune responses when using the ThyAdMSCs for cell therapy [24,25]. The use of human platelet lysate requires the addition of heparin due to residual fibrinogen [26]. Opinions differ on the concentration of heparin required to prevent coagulation without inhibiting MSC expansion. Hemeda and colleagues concluded in their study that a limit of 0.61 IU/mL of unfractionated heparin should be added [27]. Others found no differences in the proliferative capacity, expression of surface markers or multilineage differentiation of MSCs when using medium containing 10% pPL and 2 IU/mL of heparin without stabiliser compared to platelet lysate, in which no heparin was added but the fibrinogen was mechanically disrupted [25]. In our preliminary studies to verify this issue, the addition of heparin sodium 25,000 at a concentration of 2 IU/mL resulted in good proliferation of ThyAdMSCs without gelling of the medium, compared to medium containing heparin sodium 5000 (2 IU/mL), which negatively affected the cell proliferation of ThyAdMSCs. When the anticoagulant fondaparinux sodium (Arixtra; Mylan, the Netherlands) was used, gelling of the medium could not be prevented, even at high concentrations. This is consistent with other reports that the addition of 2 IU/mL heparin for MSC culture has no negative effects [28,29]. Attempts are also being made to produce human platelet lysate without heparin [25,30]. Despite the complications with pPL mentioned above, the use of pPL for cell culture has become established, replacing the previously used animal serum as a growth factor supplier, as it has been shown to be safe and without xenogeneic-related complications. Furthermore, it leads to rapid and effective ex vivo MSC expansion, which is essential for cell therapy applications, without compromising the immunosuppressive functionality of MSCs [31].

This desired immunomodulatory effect of MSCs could also be demonstrated for ThyAdMSCs under the culture conditions described in this study. When freshly isolated and activated PBMCs from healthy donors were exposed to ThyAdMSCs, the proliferation of activated PBMCs was significantly impaired by ThAdMSCs, regardless of the medium or isolation technique used. The high cytokine production of MSCs in the presence of activated PBMCs leads to an inhibition of immune cell proliferation [32]. The reduced IDO production in peripheral blood cells in the presence of ThyAdMSCs also reflects the immunomodulatory properties of ThyAdMSCs [33,34].

There are still several steps to be taken before adult thymic MSCs can be used for clinical cell therapy. Currently, allogeneic MSCs are preferred over autologous MSCs because allogeneic MSCs are safe to use and the cells can be isolated and donated from healthy volunteers, which may be advantageous compared to cells from elderly patients or those with pre-existing diseases [35]. In our study, we found no difference in cell proliferation, surface marker expression, and multilineage differentiation capacity of isolated ThyAdMSCs despite large differences in age, BMI, or pre-existing disease of the tissue donor. Further studies are needed to clarify the role of ThyAdMSCs in autologous regenerative therapy; for example, in post-MI cardiac repair or in cases of DSWI [36,37,38]. As MSCs exert their effects via paracrine signalling rather than direct engraftment and cell replacement, extracellular vesicles (EVs) derived from mesenchymal stromal cells or the secretome are also of great interest [20,39].

## 5. Conclusions

The present study demonstrates a potential use of adult thymus-derived MSCs for regenerative therapy in cardiac surgery, as they can be isolated and expanded ex vivo in sufficient numbers. Furthermore, they showed immunomodulatory effects in MLR and ELISA. The results also show that further efforts are needed to investigate their use in ischaemic heart disease and wound healing in more detail. Future work will focus on the preconditioning of ThyAdMSC cultures [40,41] and the isolation of ThyAdMSC-derived exosomes [39].

## Figures and Tables

**Table 1:** Flow cytometry antibodies.

Name	RRID *	Source
CD105-PerCP-Cy5.5	AB_2033933	BD Bioscience
CD90-FITC	AB_395969	BD Bioscience
CD73-APC	AB_10612019	BD Bioscience
CD31-APC-Cy7	AB_2738350	BD Bioscience
SSEA4-V450	AB_10896140	BD Bioscience
CD271-PE-Cy7	AB_10894762	BD Bioscience
	Cat #	Source
PE-hMSC negative cocktail	51-9007661	BD Bioscience
PE-hMSC isotype control negative cocktail	51-9007662	BD Bioscience
Stain Buffer (FBS)	554656	BD Bioscience

* RRID: Research Resource Identifier; # Order number.

## Data Availability

The data, method protocols, and information on materials used are available on request from Martina Ramsperger-Gleixner for all interested researchers: martina.ramsperger-gleixner@uk-erlangen.de.

## Abbreviations

The following abbreviations are used in this manuscript:

CD31	Platelet endothelial cell adhesion molecule (PECAM-1).
CD73	Ecto-5′-nucleotidase (NT5E).
CD90	Thymocyte differentiation antigen 1 (Thy-1).
CD105	Endoglin (ENG).
CD271	Low affinity nerve growth factor receptor (LNGFR).
αMEM	Alpha Minimal Essential Medium.
DMEM	Dulbecco’s Modified Eagle’s Medium.
IDO	Indolamine-2:3-dioxygenase.
pPL	Pooled human platelet lysate.
SSEA4	Stage specific embryonal antigen 4.
ThyAdMSC	Thymic adipose-derived mesenchymal stromal cell.
